# Toll-like receptor mediated activation is possibly involved in immunoregulating properties of cow's milk hydrolysates

**DOI:** 10.1371/journal.pone.0178191

**Published:** 2017-06-08

**Authors:** M. B. Gea Kiewiet, Renske Dekkers, Marjan Gros, R. J. Joost van Neerven, Andre Groeneveld, Paul de Vos, Marijke M. Faas

**Affiliations:** 1 Immunoendocrinology, Division of Medical Biology, Department of Pathology and Medical Biology, University Medical Center Groningen, University of Groningen, Groningen, The Netherlands; 2 FrieslandCampina, Amersfoort, The Netherlands; 3 Department of Obstetrics and Gynecology, University of Groningen, University Medical Center Groningen, Groningen, the Netherlands; University of Colorado Boulder, UNITED STATES

## Abstract

Immunomodulating proteins and peptides are formed during the hydrolysis of cow’s milk proteins. These proteins are potential ingredients in functional foods used for the management of a range of immune related problems, both in infants and adults. However, the mechanism behind these effects is unknown. We hypothesize that the interaction of peptides with Toll-like receptors (TLRs) can induce immune effects, since these receptors are known to sample many dietary molecules in the intestine in order to regulate immune effects. To investigate this, we compared the immune effects and TLR activation and inhibition by whey and casein hydrolysates with different hydrolysis levels. We first measured cytokine production in primary peripheral blood mononuclear cells stimulated with either whey, casein, or their hydrolysates. IL-10 and TNFα were induced by whey hydrolysates (decreasing with increasing hydrolysis level), but not by casein hydrolysates. Next, the activation of TLR 2, 3, 5 and 9 receptors were observed by intact and mildly hydrolysed whey proteins only and not by casein hydrolysates in TLR reporter cell lines. Many casein hydrolysates inhibited TLR signaling (mainly TLR 5 and 9). These results demonstrate that the effects of cow’s milk proteins on the immune system are protein type and hydrolysis dependent. TLR signaling is suggested as a possible mechanism for differences in effect. This knowledge contributes to a better understanding of the immune effects of hydrolysates and the design of infant formula, and nutrition in general, with specific immunoregulatory effects.

## Introduction

Many aspects of the immune system of newborns are not yet fully developed. The numbers of antigen presenting cells are for example lower, and costimulatory molecules are less expressed in newborns as compared to adults [1]. Also, Th1 responses and B cell differentiation are attenuated in newborns as compared to adults [2]. Because of this, infants have a higher risk of infections than adults [3]. Specific proteins and carbohydrates in breast milk guide and facilitate the maturation of the immune system of newborns [4]. In those cases where breast milk is not a feasible option, children are fed with infant formula produced from cow’s milk. Recently, a number of studies demonstrated that not only human mother milk but also bovine milk contains proteins that are able to actively contribute to immune development [5,6]. Immune modulating activity was found in peptides that were formed by hydrolysis of cow’s milk proteins during the digestion process [7,8]. The bioactive peptides formed have been demonstrated in the jejunum, duodenum, ileum, and even in the plasma of humans after dairy consumption [7,9,10]. These insights led to the hypothesis that specific immunomodulatory peptides from cow’s milk can be used to enhance immune maturation in infants. In a broader sense, these peptides could be used to manage a range of immune related problems in both infants and adults. Therefore, several groups, including ours, propose to develop nutritional products enriched with immunomodulatory peptides.

Hydrolyzation of cow’s milk proteins, both whey and casein fractions, results in a mixture of different peptide-sequences which is called a hydrolysate. Hydrolysates are already being produced on a large scale, mainly for the use of managing cow’s milk allergy in infants. Protein-derived peptides formed during hydrolysis have been shown to modulate the gastrointestinal tract at different levels, besides preventing IgE induced allergic responses. Immunoregulatory peptides found in cow’s milk hydrolysates were shown to induce mucus production [11,12] and to promote anti-inflammatory responses in intestinal epithelial cells [13–15]. Once taken up into the lamina propria, hydrolysates may also directly influence both innate and adaptive immune cells [16–18]. Macrophages showed both inhibition of inflammatory responses [16,19] and an increased phagocytosis capacity after incubation with casein hydrolysates *in vitro* [17,20,21] and *in vivo* [22,23]. Both stimulatory and inhibitory effects were observed on lymphocyte proliferation after adding whey and casein hydrolysates [18,24,25]. Furthermore, cow’s milk hydrolysates induced differentiation of specific T cell subsets, especially the formation of interleukin-10 (IL-10) producing regulatory T cells, in cultured human lymphocytes [26], and in mice [27].

Although many immunoregulatory effects of hydrolysates have been described, the underlying mechanisms to explain the aforementioned effects are still unknown. Toll-like receptors (TLRs) play an important role in intestinal immune regulation [28]. TLRs are a family of pathogen recognition receptors (PRRs) that serve as sensors for the immune system. TLRs are expressed on most immune cells, including epithelial cells, dendritic cells, and lymphocytes [29–31]. PRRs are responsible for modulation of the intestinal immune response by interacting with pathogen associated molecular patterns and with food associated molecules in the lumen. Previous studies show immune regulating properties of dietary fibers through TLRs, including sugars and starches [32–34]. TLRs are known to bind a variety of ligands such as lipids, proteins, and nucleic acids [35,36]. Although data are limited, recent studies also suggest that whey proteins can affect immune responses via TLRs [37]. To gain more insights in this capacity of cow’s milk proteins, in the present study we tested whether different whey and casein hydrolysates are able to signal via TLRs. To this end we tested the TLR activating and inhibiting capacity for TLR 2, 3, 4, 5, 7, 8 and 9 of a range of cow’s milk hydrolysates.

## Materials and methods

### Ethical statement

For blood sampling of human volunteers, written informed consent was obtained. Data were analyzed and presented anonymously. The present research and consent procedure was approved by the ethical review board of the University Medical Center Groningen, as stated in the application ‘‘2007/255”. All clinical investigation was conducted according to the principles stated in the Declaration of Helsinki.

### Test materials

All materials tested (hydrolysates and intact proteins) were kindly provided by FrieslandCampina (Amersfoort, the Netherlands). All intact proteins (casein or whey protein) were hydrolysed by the use of enzymes (peptidases, proteases) for a certain time period. Upon heat inactivation of the enzymes, a filtration step was incorporated before drying. Both whey and casein hydrolysates with a degree of hydrolysis (DH) ranging from 3.1% to 45% (Table 1) were tested. In all experiments, intact whey and casein served as controls. All samples were tested for endotoxins by means of the Limulus amebocyte lysate assay (LAL) [38]. Since proteins can interfere with LAL [39], lipopolysaccharide (LPS) and peptidoglycan (PG), which are major bacterial-derived contaminants in commercially available biological substances [40], were quantified separately by ELISAs (BlueGene, Shanghai, China). The concentrations measured have no influence on the cells applied in this study.

**Table 1:** Overview of the characteristics of the studied hydrolysates.

Hydrolysate/Sample	Source	Degree of hydrolysis (DH)
A	Whey: WPC80	3.1%
B	Whey: WPI90	6%
C	Whey: WPC80	7%
D	Whey: WPC80	9.2%
E	Whey: WPC80	15%
F	Whey: WPC80	22%
Whey	WPC80	0%
G	Acid casein (90% casein)	7%
H	Acid casein (90% casein)	13%
I	Acid casein (90% casein)	17%
J	Acid casein (90% casein)	21%
K	Acid casein (90% casein)	37%
L	Acid casein (90% casein)	45%
Casein	Caseinate (90% casein)	0%

### Primary PBMC isolation, stimulation, and cytokine (IL-8, IL-10, TNFα) measurement

Primary PBMCs were isolated from venous blood from 3 healthy, adolescent, male volunteers. Blood was collected in heparinized tubes (15 IU/ ml lithium-heparin, Becton Dickinson B.V., Breda, The Netherlands). Subsequently primary PBMCs were isolated by Ficoll density gradient separation according to the manufacturer’s protocol (Lymphoprep, Axis-Shield, Oslo, Norway). Primary PBMCs were seeded in a 96 wells round bottom plate at a density of 0.4x10^6^ cells/well, in a volume of 200 μl medium, and stimulated with 2 mg/ml hydrolysate for 24 hours (37°C, 95% oxygen, 5% CO_2_). We selected 2 mg/ml based on dose response curves performed in THP-1 and HEK-Blue human TLR2 cells (S1 and S2 Figs). This concentration increased TLR activation significantly and more than two-fold for all TLR activating samples in both cell lines. A combination of phorbol myristate acetate (PMA; Sigma Aldrich, Zwijndrecht, the Netherlands) (5 ng/ml) and ionomycin (500 ng/ml) was used as a positive control. Unstimulated cells served as a negative control. All stimulations were performed in technical triplicates. After 24 hours, supernatant was collected and stored at -80°C. To analyze cytokine levels a self-composed multiplex immunoassay was used containing ProcartaPlex Simplexes for the human anti-inflammatory cytokine IL-10, and the pro-inflammatory cytokines IL-8, and tumor necrosis factor alpha (TNFα) (Affymetrix, Santa Clara, USA). The immunoassay was performed according to the manufacturer’s protocol. Briefly, cytokine standards of the different Simplexes were mixed, and serial dilutions were prepared, followed by mixing antibody magnetic beads and aliquoting the mixture for analysis. After washing, standards and samples were added (50 μl/well), the plate was sealed, and incubated while shaking (30 minutes at room temperature (RT), overnight at 4°C, and again 30 minutes at RT). After washing the plate twice, detection antibodies were added (25 μl/well) and the plate was incubated for 30 minutes at RT on a plate shaker. After incubation the plate was washed twice and 50 μl/well streptavidin-phycoerythrin was added. Again, the plate was incubated at RT for 30 minutes while shaking. To prepare the plate for analysis, the plate was washed, and 120 μl/well of reading buffer was added. After shaking the plate for 5 minutes at RT fluorescence was measured using a Luminex 100 System. The data obtained were analyzed using StarStation software. The mean and SD of the data were plotted as the fold change compared to the positive control, which was set to 1.

### Culturing of THP-1 and HEK reporter cell lines

To determine the effects of hydrolysates on TLR signaling, various THP-1 and HEK reporter cell lines, all purchased from InvivoGen (Toulouse, France), were used. In order to quantify TLR activation in the cells, all cell lines contained a construct for Secreted Embryonic Alkaline Phosphatase (SEAP), which was coupled to the nuclear factor kB/Activating protein-1 (NF-kB/AP-1) promoter. NF-κB/AP-1 is a well-known downstream target of TLR receptors [32,41]. To assess the TLR dependent effects of cow’s milk hydrolysates, two THP-1 human acute monocytic leukemia cell lines were used which express all TLRs endogenously. The first THP-1 cell line (THP1-XBlue^™^-MD2-CD14) expressed MD2 and CD14, thus responds to TLR ligands. To check for TLR dependency, the results obtained with the THP-1 cell line were compared with a second THP-1 cell line (THP1-XBlue^™^-defMyD). This cell line expresses a truncated, non-functional form of the TLR adapter Myeloid differentiation primary response gene 88 (MyD88), and is therefore unresponsive to TLR2, 4, 5, 6, 7, 8, and 9 activation. THP-1 cell lines were cultured in RPMI1640 medium (Gibco, Life Technologies, Bleiswijk, The Netherlands), containing 10% heat inactivated FBS (Fetal Bovine Serum, HyClone, Thermo Scientific, Breda, The Netherlands), 2 mM L-glutamine, 1.5 g/L sodium bicarbonate (Boom B.V. Meppel, The Netherlands), 4.5 g/L glucose, 10 mM HEPES, 1.0 mM sodium pyruvate, 100 mg/ml Normocin^™^ (Invivogen, Toulouse, France), and 50 U/ml and 50 μg/ml Penicillin/Streptomycin. All additives were purchased from Sigma Aldrich (Zwijndrecht), unless indicated otherwise. Both THP-1 cell lines were passaged twice a week by inoculating 5x10^5^ cells.

To study the effects on individual TLRs, 7 Human embryonic kidney (HEK)293 cell lines (HEK-Blue^™^-hTLRX) were used, each containing an inserted construct of either human TLR2, 3, 4, 5, 7, 8, or 9. HEK cells were cultured in DMEM medium (Gibco, Life Technologies, Bleiswijk, The Netherlands), supplemented with 10% heat inactivated FBS, 2 mM L-glutamine, 4.5 g/l glucose, 50 U/ml and 50mg/ml penicillin/streptomycin, and 100 mg/ml Normocin. These HEK cells were grown to ~80% confluency. All reporter cell lines were cultured for 3 passages before they were maintained in zeocin containing selection media (InvivoGen, Toulouse, France) as previously described [32,41].

### Reporter cell stimulation assays and Quanti-Blue analysis

All relevant ligands were purchased from InvivoGen (Toulouse, France) (Table 2). THP-1 cells were collected by centrifuging (5 minutes, 1500 rpm), and resuspended (Table 2) in culture medium. Cells were seeded in a flat bottom 96 wells plate (100 μl/well), and stimulated for 24 hours (37°C, 95% oxygen, 5% CO_2_) with 2 mg/ml hydrolysate, intact protein, or a relevant ligand as a positive control (Table 2). Medium was used as a negative control. After incubation, Quanti-Blue detection medium was used to analyze the cell supernatant as described before [32]. Absorbance (650 nm) was measured using a VersaMax microplate reader (Molecular Devices GmbH, Biberach an der Riss, Germany) and SoftMax Pro Data Acquisition & Analysis Software to determine SEAP activity, which represents activation of NF-κB/AP-1. Assays using HEK cells were performed in a similar way, and as described before [32,41]. HEK cells were detached by tapping the flask, and resuspended according to the manufacturer’s protocols (Table 2). A cell suspension of 180 μl was added in each well of a flat bottom 96 wells plate, and cells were stimulated for 24 hours (37°C, 95% oxygen, 5% CO_2_) with 2 mg/ml hydrolysate. For each HEK cell line a corresponding TLR ligand was used as a positive control (20 μl) (Table 2), medium was used as a negative control. After 24 hours, SEAP activity was measured as described above.

**Table 2:** Cell densities and ligands used in the different reporter cell line assays.

Cell line	Cell density for seeding	Positive control (concentration in well)
THP-1 MD2-CD14	1*10^6^ cells/ml	*Escherichia coli K12* Lipopolysaccharide (10 ng/ml)
THP-1 MyD88 deficient	2*10^6^ cells/ml	MurNAc-L-Ala-γ-D-Glu-mDAP (M-TriDAP, 100 μg/ml)
HEK-Blue human TLR2	2.8*10^5^ cells/ml	Heat killed Listeria monocytogenes (10^7^ cells/ml)
HEK-Blue human TLR3	2.8*10^5^ cells/ml	Polyinosinic-polycytidylic acid (high molecular weight) (5 μg/ml)
HEK-Blue human TLR4	1.4*10^5^ cells/ml	*Escherichia coli K12* Lipopolysaccharide (10 ng/ml)
HEK-Blue human TLR5	1.4*10^5^ cells/ml	*Salmonella typhimurium derived* flagellin (10 ng/ml)
HEK-Blue human TLR7	2.2*10^5^ cells/ml	Imiquimod (5 mg/ml)
HEK-Blue human TLR8	2.2*10^5^ cells/ml	Single stranded RNA (ssRNA40/LyoVecTM, 2 μg/ml)
HEK-Blue human TLR9	4.5*10^5^ cells/ml	Type B CpG oligonucleotide (ODN 2006, 0,25 μM)

The mean and SD for each sample were plotted as the fold-change compared to the negative control, which were unstimulated cells. The negative controls were set at 1.

### HEK reporter cell inhibition assays

Besides activating TLRs, TLR binding molecules can also inhibit pattern recognition receptors [33]. To assess inhibition of the TLR signaling by hydrolysates, HEK cells were resuspended, and seeded in the same way as described above for the stimulation assays. Then, cells were stimulated with the appropriate TLR ligand (Table 2), together with 2 mg/ml hydrolysate. The TLR ligand alone served as a positive control, medium alone as a negative control. Cells were incubated for 24 hours (37°C, 95% oxygen, 5% CO_2_), and SEAP activity was measured as described above. The mean and SD for each sample were plotted as the fold-change compared to the positive control, which were cells stimulated with its relevant ligand. The positive controls were set at 1.

### NF-κB dependency of cytokine production in THP-1 macrophages

In order to investigate the role of NF-κB in whey hydrolysate induced cytokine production, THP-1 monocytes were first differentiated into THP-1 macrophages as previously described [42]. First, THP-1 monocytes were cultured in RPMI medium (Lonza, Verviers, Belgium) supplemented with 10% decomplemented FBS, 2mM L-glutamine (Lonza, Verviers, Belgium), 1mM sodium pyruvate (Lonza, Verviers, Belgium), 0.05mM 2-mercaptoethanol (Scharlau, Barcelona, Spain), 60 μg/ml gentamycin sulfate (Lonza, Verviers, Belgium), and 2.2 μg/ml amphotericin B (Sigma Aldrich, Zwijndrecht, the Netherlands). Then, cells were seeded in a 24 wells plate at a concentration of 1x10^6^ cells/well (in 0.5 ml medium). To differentiate the cells into macrophages 100 ng/ml PMA was added. After 48 hours, the PMA was removed and cells were washed twice with fresh medium. Cells were cultured for another 24 hours. Then, cells were incubated with either 10 μM of the NF-κB inhibitor celastrol (Invivogen, Toulouse, France) or medium (control) for 30 minutes. Next, 2 mg/ml intact whey protein or its hydrolysates were added. Cells were incubated for 24 hours, after which the supernatant was collected. TNFα and IL-10 levels were measured in the supernatant by ELISA according to the manufacturer’s protocol (eBioscience, San Diego, USA).

### Statistical analysis

Statistical analysis was performed using Graphpad Prism. Normal distribution of the data was tested using the Kolmogorov-Smirnov test. Values are expressed as mean ± standard deviation (SD). Significance levels of PBMC and reporter cell line data were assessed using the Kruskal-Wallis test followed by the Dunn’s test to show individual differences. Significance levels of THP-1 macrophage data was assessed by paired T-tests. A *p*-value of <0.05 was considered to indicate a significant difference.

## Results

### Intact whey and casein as well as several hydrolysates induce cytokine production in primary PBMCs

**Fig 1:**
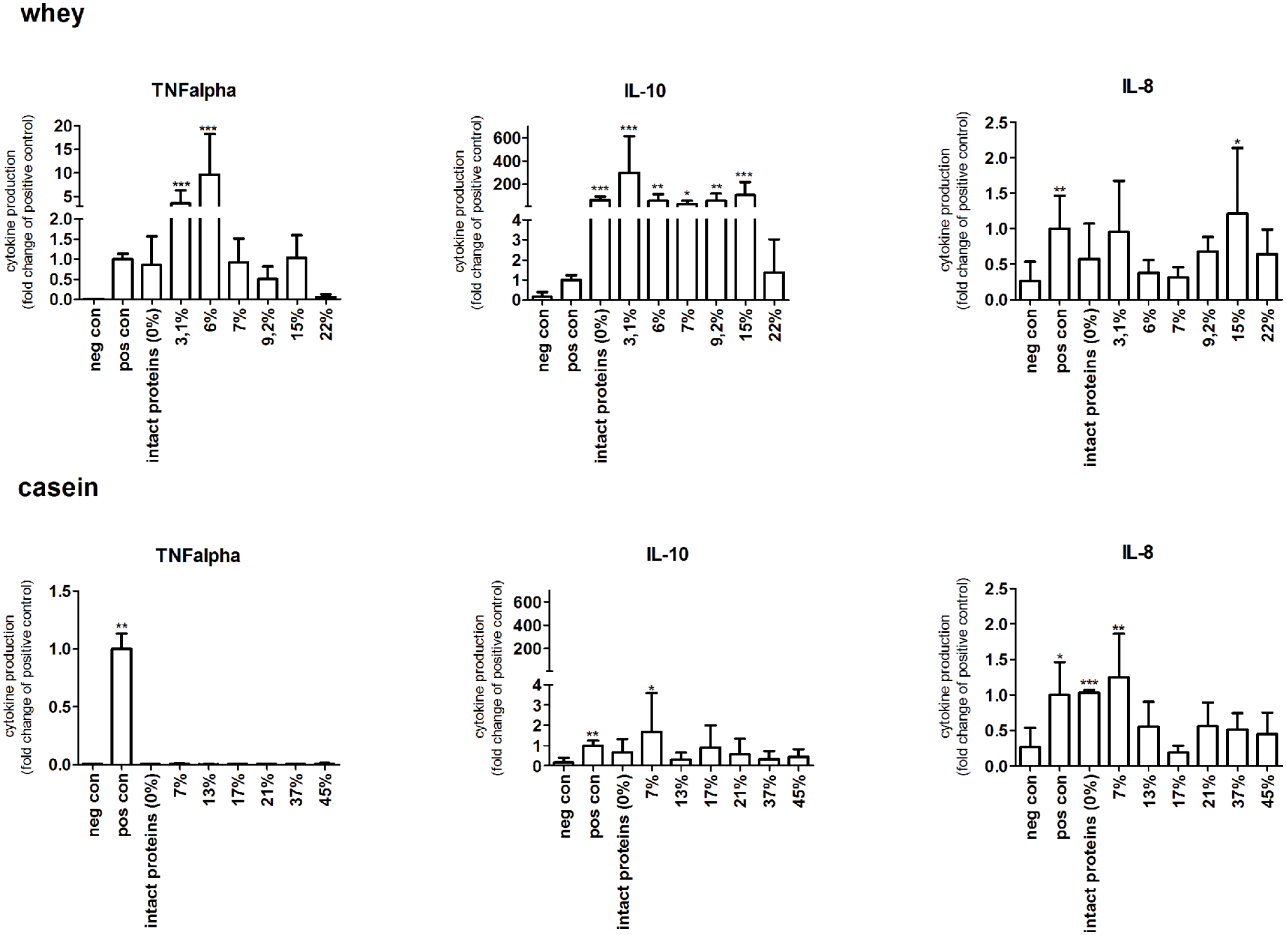
Human primary PBMCs produced cytokines after stimulation with intact whey and casein proteins or hydrolysates. Whey samples induced TNFα and IL-10 in a degree of hydrolysis dependent way. Not many effects were observed for IL-8 (upper panel). Effects induced by casein samples was less compared to the whey samples. No TNFα and IL-10 were produced after stimulation with intact casein or its hydrolysates. IL-8 production was increased by some casein samples (lower panel). Significant differences compared to the negative control were determined by using the Kruskal-Wallis test followed by the Dunn’s test and indicated by *.

The induction of pro- and anti-inflammatory cytokines in human primary PBMCs was tested after incubation with 2 mg/ml hydrolysate, or intact protein for 24 hours. To gain insight in the anti-inflammatory response IL-10 was measured followed by TNFα and IL-8 to suggest pro-inflammatory effects (Fig 1).

First whey proteins were studied. As shown in Fig 1, intact whey proteins increased TNFα production to a level comparable to the positive control, although not significant. The two hydrolysates with the lowest DH, 3.1% and 6%, induced high levels of TNFα secretion (*p*<0.001). Further hydrolyzed proteins induced lower levels of TNFα similar to the effect of intact whey. When whey proteins were hydrolyzed extensively, up to a DH of 22%, the TNFα producing capacities of the proteins was lost.

The production of IL-10 after stimulation with whey protein and hydrolysates was high. Intact whey protein (*p*<0.001) and all whey hydrolysates (between *p*<0.05 and 0.001) were able to induce high IL-10 production, except for the most extensively hydrolyzed whey sample, which did not significantly increase IL-10 production. The least hydrolyzed whey sample, with a DH of 3.1%, increased IL-10 levels most (*p*<0.001). After stimulation with intact whey protein or whey hydrolysates some production of IL-8 was observed, but the increase was low compared to the other cytokines. Only the hydrolysate with a DH of 15% significantly induced IL-8 production (*p*<0.05).

For casein the effects on primary PBMC cytokine production were mostly lower than for whey. Stimulation of human primary PBMCs with intact casein did not induce increased TNFα production. Also none of the casein hydrolysates, including the mildly hydrolyzed casein, induced significant levels of TNFα.

IL-10 levels were also not increased after stimulation of primary PBMCs with intact casein. Furthermore, none of the hydrolysates induced significantly increased levels of IL-10 compared to the negative control. IL-8 production was mildly increased by intact casein and some hydrolysates. Intact casein significantly increased IL-8 production (*p*<0.001). The only hydrolysate that was able to induce IL-8 expression was characterized by a DH of 7% (*p*<0.05). These data suggest that intact whey and casein as well as whey hydrolysates are able to induce the most pronounced immune effects.

### Intact proteins and specific whey hydrolysates induce TLR activation

The above mentioned induction of cytokine production by hydrolysates, prompted us to determine whether pattern recognition receptors may be involved in the underlying mechanisms of the differences observed in cytokine-release. We therefore applied a technology platform of pathogen recognition receptor expressing cell lines [32,41].

To determine possible TLR stimulating capacity of whey, casein and their hydrolysates, samples were tested on a THP-1 reporter cell line [32,41]. Next, TLR dependency of the observed activation was determined by testing the effects of the hydrolysates on the THP-1 reporter cell line carrying a truncated TLR adapter molecule MyD88. A response in the THP-1 MD2-CD14 and an absence or suppressed responses in THP-1 MyD88 deficient indicates involvement of TLRs in the responses.

**Fig 2:**
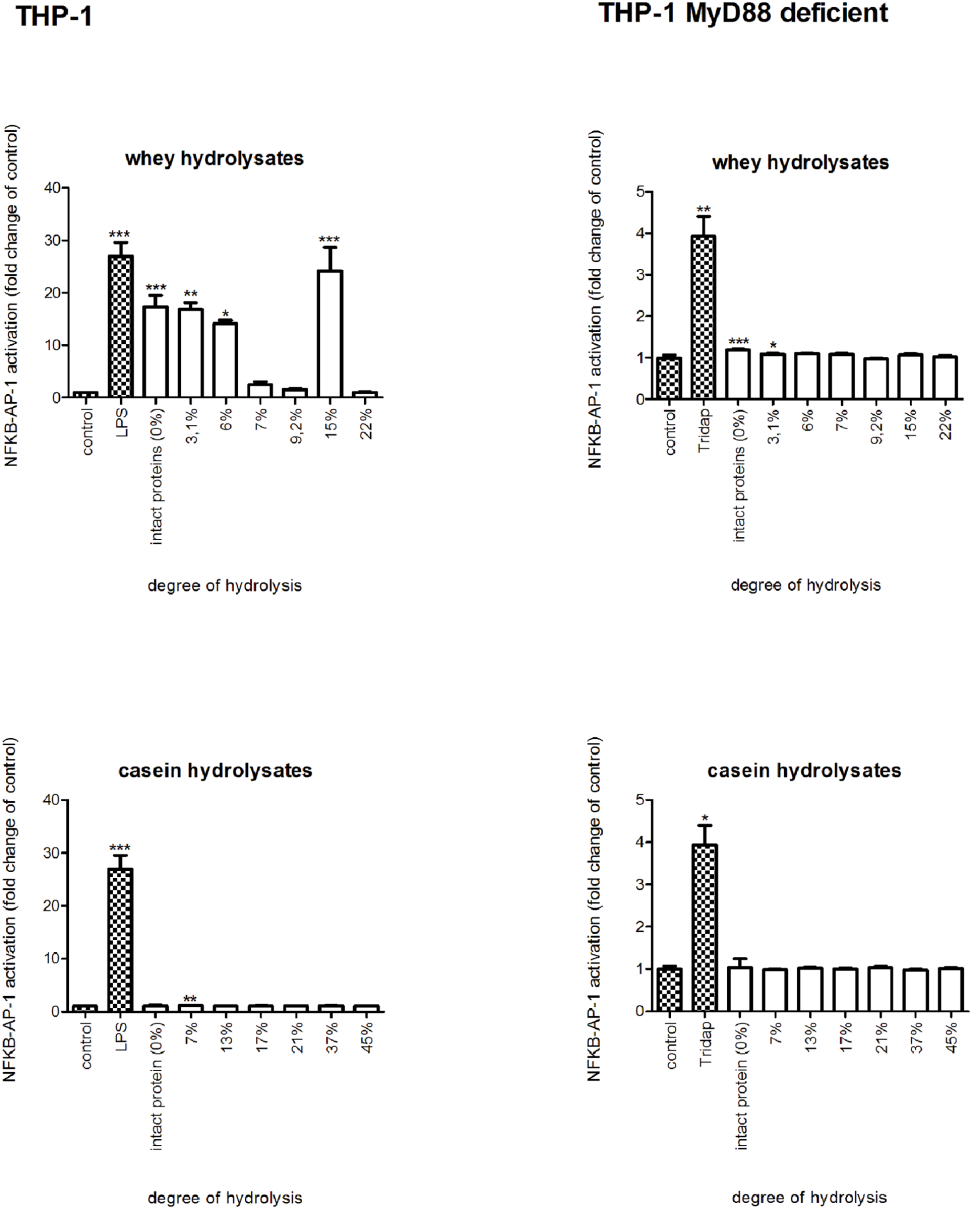
NF-kB/AP-1 expression in THP-1-MD2-CD14 and THP-1-MyD88 deficient reporter cells after stimulation with intact whey and casein proteins or hydrolysates. Intact whey and the two most mildly hydrolyzed whey hydrolysates induced TLR signaling, while casein samples had no effect (left panel). These effects were TLR dependent, since no activation was observed in the MyD88 deficient cell line after stimulation (right panel). Significant differences compared to the negative control were determined by using the Kruskal-Wallis test followed by the Dunn’s test and indicated by *.

Intact whey protein had a profound activating effect (*p*<0.001) on TLR signaling in THP-1 cells. The two most mildly hydrolyzed samples with DH 3.1% and 6% induced a statistically significant increase (*p*<0.05 and 0.01), which is similar to the activation level of the intact whey proteins. For the other hydrolysates TLR activation was low compared to the more mildly hydrolyzed samples, and was not significant compared to the negative control. However, the hydrolysate with a DH of 15% was an exception, and showed a significant TLR activation (*p*<0.001). The extensively hydrolyzed samples did not induce TLR activation. The effects were protein type specific, since intact casein did not induce TLR activation. Furthermore, also none of the casein hydrolysates showed TLR activation. By comparing the left (THP-1 reporter cell line) and right part (THP-1 reporter cell line, MyD88 deficient) of Fig 2 it has to be concluded that the majority of the responses in the THP-1 reporter cells induced by whey protein and it’s hydrolysates are MyD88 and thus TLR-dependent.

### Hydrolysis changed TLR type specific activating properties of whey proteins

**Fig 3:**
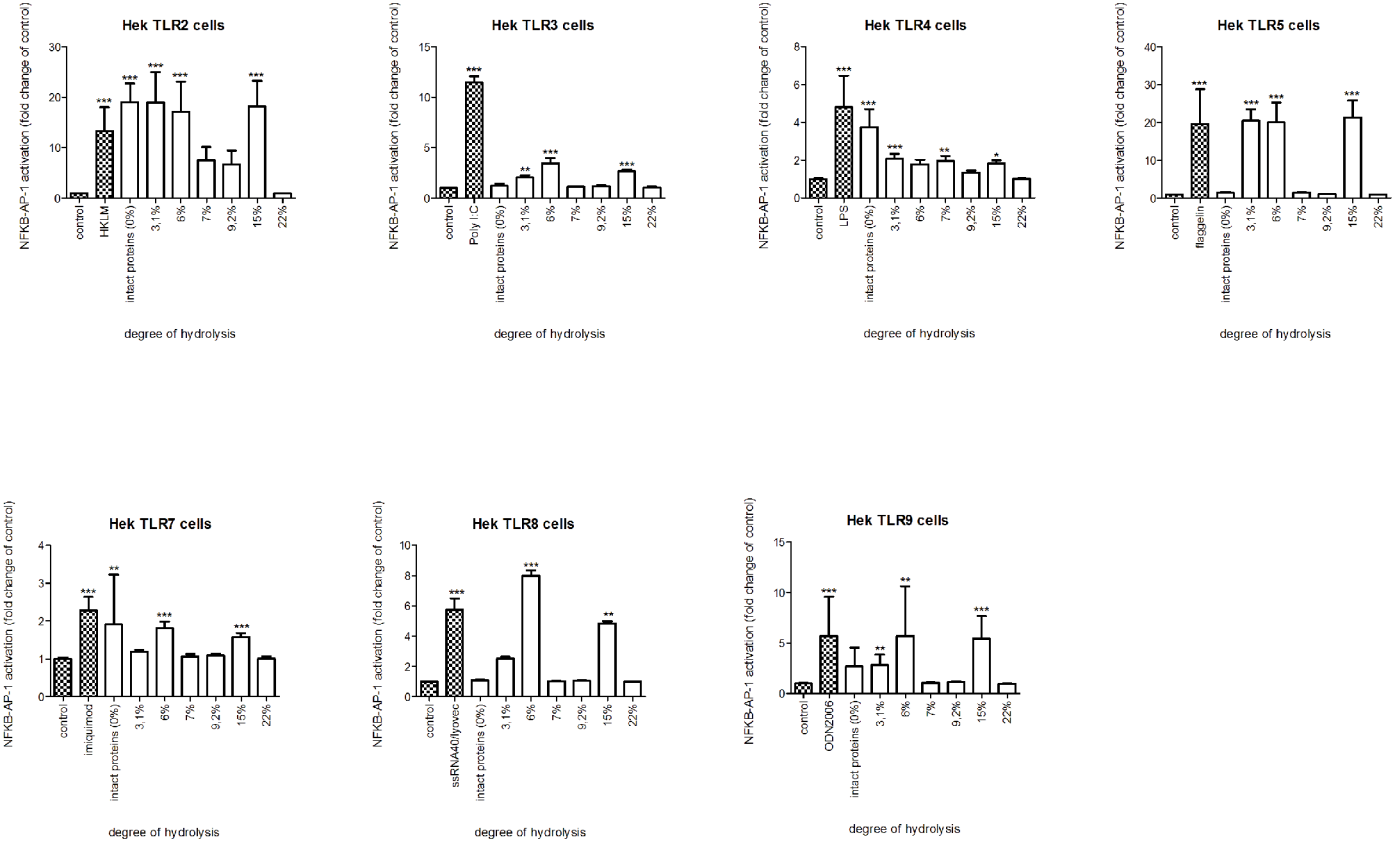
NF-kB/AP-1 expression in HEK reporter cells carrying individual TLRs after stimulation with intact whey protein or hydrolysate. Intact whey protein induced signaling of TLR 2, 4, and 7, but this pattern of TLR activation by whey changed after hydrolysis. The TLR activating capacity of whey hydrolysates decreased with increasing level of hydrolysis. Significant differences compared to the negative control were determined by using the Kruskal-Wallis test followed by the Dunn’s test and indicated by *.

**Fig 4:**
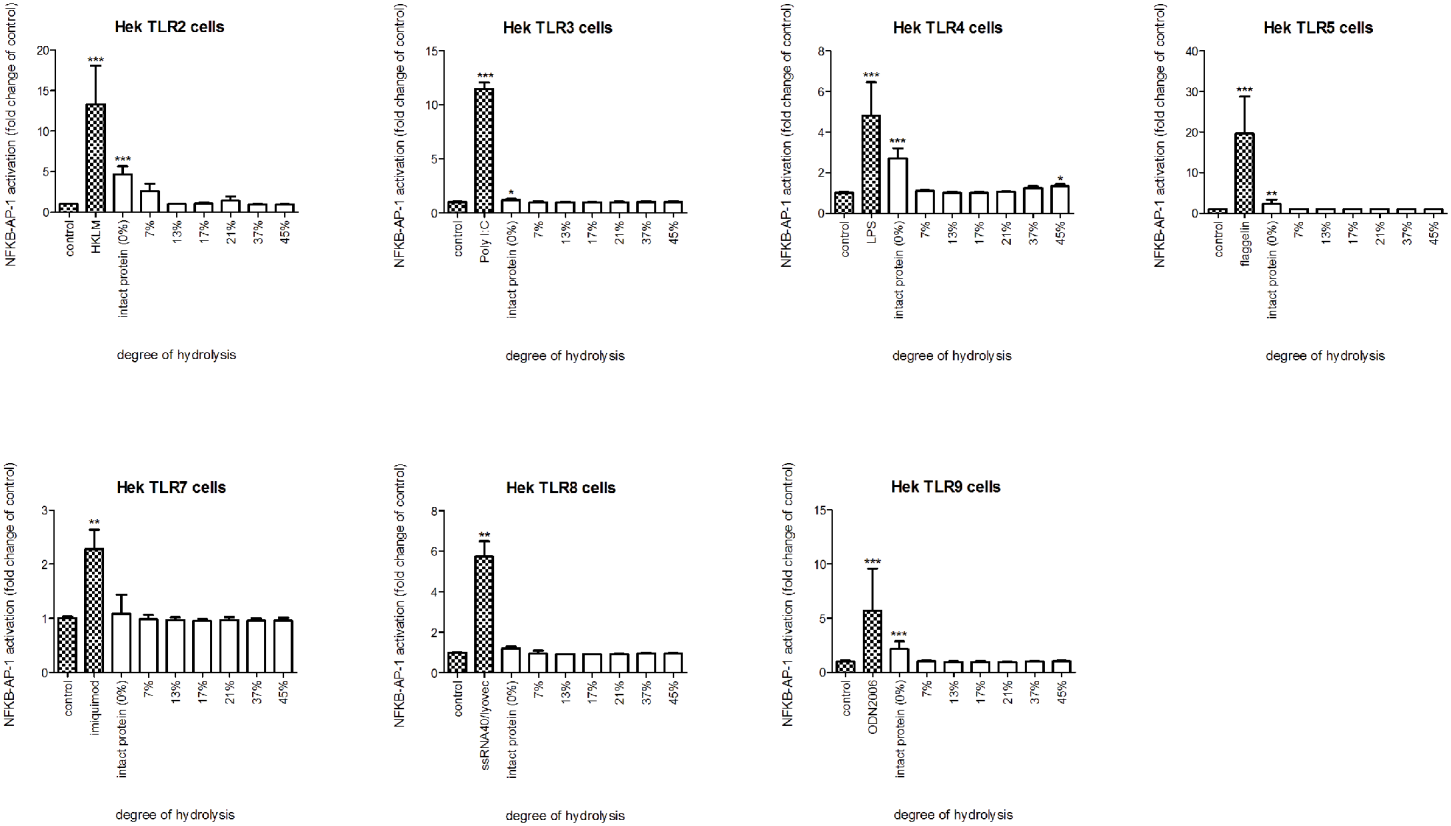
NF-kB/AP-1 expression in HEK reporter cells carrying individual TLRs after stimulation with intact casein protein or hydrolysate. Intact casein stimulated TLR2, 3, 4, 5 and 9. The TLR activating capacity was completely lost after more extensive hydrolysis. Significant differences compared to the negative control were determined by using the Kruskal-Wallis test followed by the Dunn’s test and indicated by *.

Next, we investigated which specific TLRs are involved in the activation of THP-1 cells. To this end, HEK reporter cell lines, each containing a construct for a specific TLR, were stimulated with intact whey and casein or its hydrolysates (Figs 3 and 4).

Intact whey protein induced activation of TLR 2, 4, and 7 (all *p*<0.001). This pattern of TLR induction by whey changed after hydrolysis. The two hydrolysates with the lowest DH significantly induced TLR activation in HEK cells expressing TLR2, 3, 5, or 9 (all *p*<0.001) but not TLR4 and 7. The hydrolysate with DH 6% also had a different induced TLR activation as it also activated TLR8 (*p*<0.001), while the hydrolysate with DH 3,1% still induced TLR4 activation (*p*<0.001).

Activation of TLRs was less in most of the hydrolysates with a higher DH. The hydrolysate with DH 9,2% only increased TLR4 (*p*< 0.01). However, the hydrolysate with a DH of 15% activated all TLRs (*p*<0.001). For the most extensively hydrolyzed whey samples, no TLR activation was observed.

Intact casein stimulated TLR2, 3, 4, 5 and 9 (TLR2, 4 and 9 *p*<0.001, TLR5 *p*<0.01, TLR3 *p*<0.05), while also the casein sample with a DH of 45% slightly activated TLR4 (*p*<0.05). No other TLR activating properties were observed for either intact casein or its hydrolysates.

### Hydrolysis changed TLR type specific inhibiting properties of casein proteins

**Fig 5:**
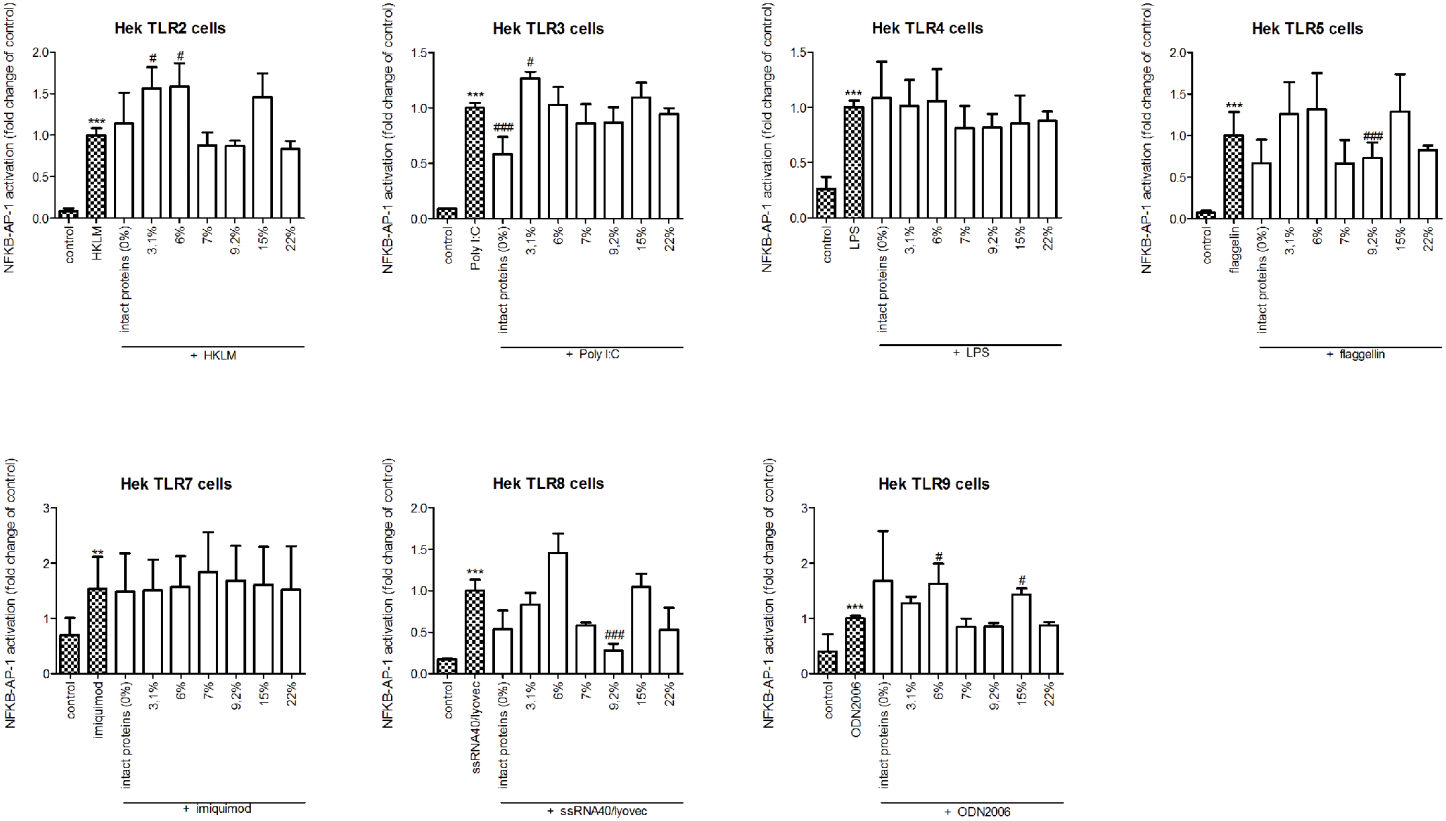
NF-kB/AP-1 expression in HEK reporter cells carrying individual TLRs after simultaneous stimulation with its relevant ligand and intact whey protein or hydrolysate. Intact whey protein significantly inhibited TLR3 activation. After hydrolysis of the proteins this effect was lost. The hydrolysate with a DH of 7% was able to inhibit TLR5 and TLR8 instead. Significant differences were determined by using the Kruskal-Wallis test followed by the Dunn’s test. Significant differences compared to the negative control were indicated by *, significant differences compared to the positive control were indicated by #.

**Fig 6:**
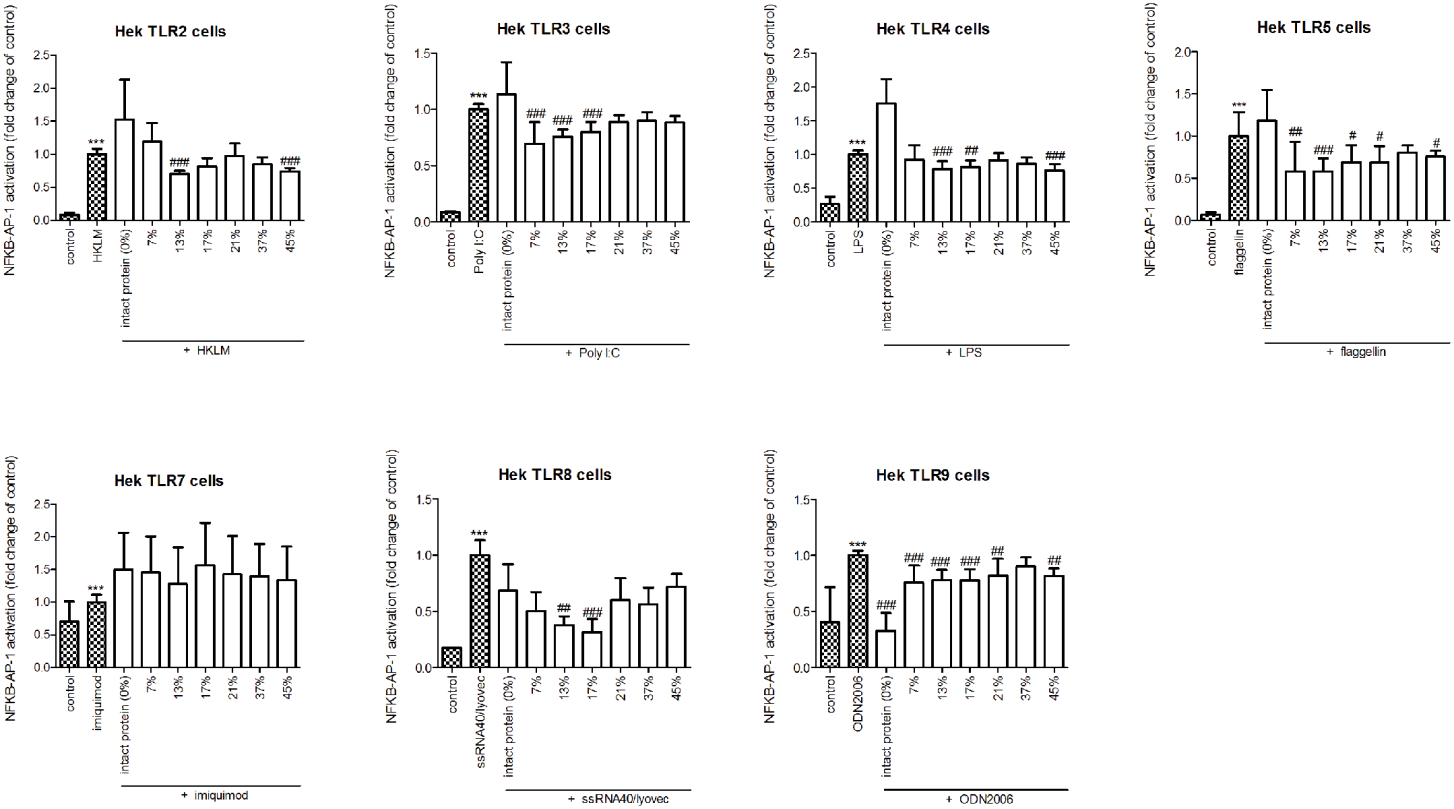
NF-kB/AP-1 expression in HEK reporter cells carrying individual TLRs after simultaneous stimulation with its relevant ligand and intact casein protein or hydrolysate. Casein samples were very efficacious in inhibiting TLR signaling. Intact casein strongly inhibited TLR 9. Hydrolysis of casein did change the magnitude of inhibition and the type of TLRs that were inhibited. Significant differences were determined by using the Kruskal-Wallis test followed by the Dunn’s test. Significant differences compared to the negative control were indicated by *, significant differences compared to the positive control were indicated by #.

Since TLR binding molecules have also been found to be able to inhibit TLR activation, it was also tested whether whey and casein hydrolysates were able to inhibit TLR expression induced by relevant TLR ligands [33]. To this end, whey and casein and its hydrolysates were added to the cells together with the relevant TLR ligand (Figs 5 and 6).

Intact whey protein significantly inhibited Poly I:C induced TLR3 activation (*p*<0.001), but did not inhibit any of the other TLRs. After hydrolysis all TLR3 inhibiting capacity of the whey protein was lost. As with activation, the TLR types that could be inhibited changed after hydrolysis depending on the DH. E.g. the sample with a DH of 7% was able to inhibit TLR5 (*p*<0.001) and TLR8 (*p*<0.001), but not TLR3. The other hydrolysates did not have inhibitory effects.

The two most mildly hydrolyzed whey samples showed no inhibitory effects on TLRs, in fact some of them even increased the agonistic effects on TLR2, 3, 5, and 9 activation. E.g. the HKLM induced activation of TLR2 increased 1.5 fold or more (both *p*<0.05) by both samples.

Casein samples were much more efficacious in inhibiting TLR signaling than whey. Intact casein strongly inhibited TLR 9 (*p*<0.001). Hydrolysis of casein did change the magnitude of inhibition and the type of TLRs that were inhibited. Even mild hydrolysis already induced an increase in inhibiting capacity, since the hydrolysate with a DH of 7% was not only able to inhibit TLR9 (*p*<0.001), but also inhibited TLR3 (p<0.001) and TLR5 (p<0.01). More extensive hydrolysis had even more effects. The hydrolysate with a DH of 21% only inhibited TLR5 (*p*<0.05) and 9 (*p*<0.01) and not TLR3 and 8. The extensively hydrolyzed sample (DH 45%) decreased TLR2, 4, 5, and 9 (TLR2 and 4 *p*<0.001, TLR5 *p*<0.05, TLR9 *p*<0.01) activation, but not TLR 3, 7 and 8. The sample with a DH of 17% inhibited TLR4 (*p*<0.01) in addition to TLR3, 5, 8 and 9 (TLR3, 8 and 9 *p*<0,001, TLR5 *p*<0.05) but lost the capacity to attenuate TLR8 activation. The extensively hydrolyzed casein with the widest inhibiting capacity had a DH of 13%. This hydrolysate inhibited TLR2, 3, 4, 5, 8, and 9 (all *p*<0.01).

### Cytokine expression was NF-κB dependent in THP-1 macrophages

**Fig 7:**
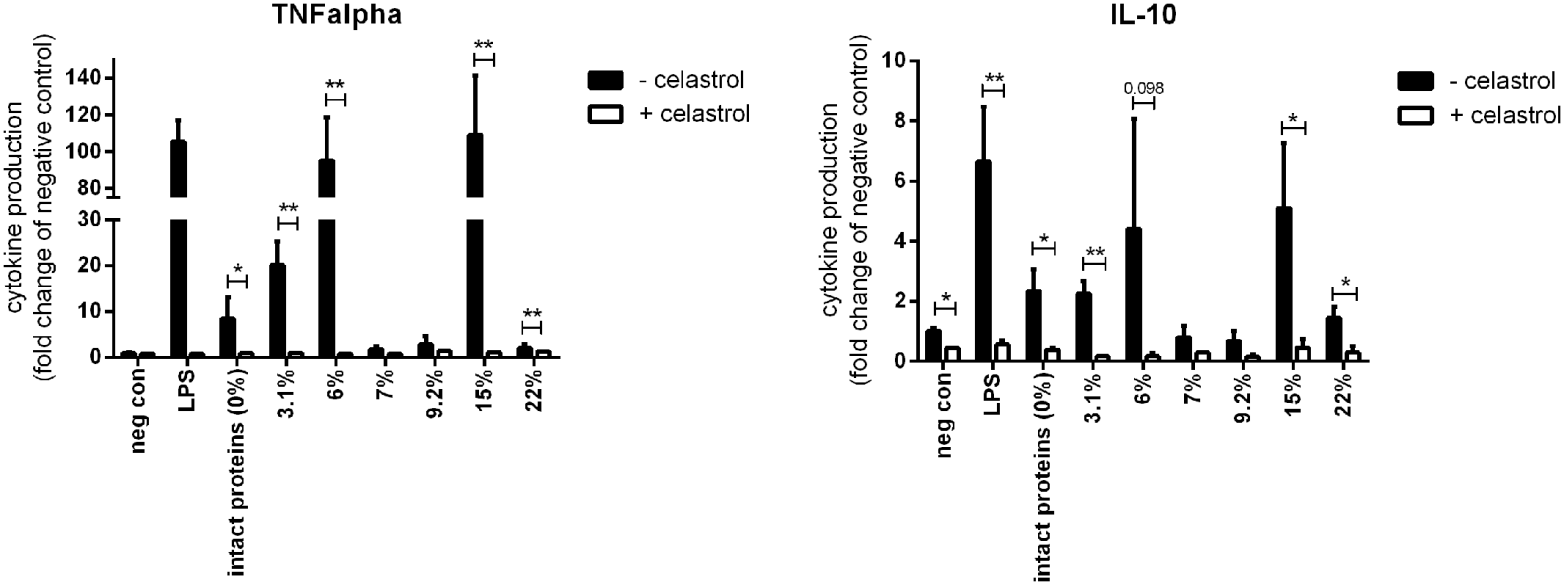
TNFα and IL-10 production by THP-1 macrophages stimulated with 2 mg/ml intact whey protein or whey hydrolysate in the presence or absence of celastrol. Celastrol significantly inhibited both TNFα and IL-10 induced by intact whey protein and whey hydrolysates, demonstrating NF-κB dependency of the cytokine production. Significant differences were determined by using paired T tests. Significant differences were indicated by *.

In order to confirm the NF-κB dependency of the cytokine expression induced by whey (hydrolysate), we stimulated PMA-differentiated THP-1 macrophages with 2 mg/ml intact whey protein or whey hydrolysate in the presence or absence of the NF-κB inhibitor celastrol. As shown in Fig 7, incubation with celastrol inhibited production of TNFα and IL-10 induced by intact whey or whey hydrolysates. TNFα production by the macrophages after exposure to intact whey (*p*<0.05) and the hydrolysates with a DH of 3.1% (*p*<0.01), 6% (*p*<0.01), 15% (*p*<0.01), and 22% (*p*<0.01) was significantly decreased by celastrol (*p*<0.01). IL-10 production induced by LPS (*p*<0.01), intact whey proteins (*p*<0.05), and hydrolysates with DH of 3.1% (*p*<0.01), 15% (*p*<0.05), and 22% (*p*<0.05) was also inhibited. Celastrol administration showed a trend towards decreasing the IL-10 production induced by the whey hydrolysate with a DH of 6% (*p* = 0.098). The hydrolysates with a DH of 7% and 9.2% did not induce TNFα and IL-10, and therefore celastrol did not have an effect. Overall, these data show the NF-κB dependency of TNFα and IL-10 production by intact whey proteins and hydrolysates.

## Discussion

To the best of our knowledge, this is the first study to compare the immunomodulatory effects of a range of cow’s milk hydrolysates varying in type and degree of hydrolysis, allowing us to study structure-effector relationships. Since TLRs are important mediators in the immune response but knowledge about the involvement of TLRs in the immunomodulatory effect of hydrolysates is still limited, immunoregulatory effects were investigated together with the TLR-activating and -inhibiting capacity of hydrolysates.

To investigate the immunomodulatory effects of the cow’s milk hydrolysates cytokine production was measured from primary PBMCs. We chose to use primary PBMCs since this population of immune cells was used before to study immunoregulatory effects of dietary molecules [32], including cow’s milk proteins [43]. Whey protein and its hydrolysates were found to possess immunomodulatory effects. Intact whey mainly induced the regulatory cytokine IL-10. As expected, hydrolysis changed the cytokine induction. Mildly (DH 3.1 and 6%) hydrolyzed whey protein stimulated not only IL-10 production, but also increased the production of the pro-inflammatory cytokine TNFα. Whey hydrolysate or a peptide fraction of this product have been shown previously to induce TNFα or IL-10 production in resting PBMCs [44] or in murine splenocytes [45]. High IL-10 levels induced by intact whey or mildly hydrolyzed whey indicate an overall anti-inflammatory effect of whey and whey hydrolysates. Anti-inflammatory effects of whey hydrolysates have indeed been described in animals using standard tests including paw edema tests [46]. Compared to whey, casein induced low levels of all cytokines measured. After hydrolysis (even mild) of casein, no cytokine production was detected which corroborates the findings of others [47,48].

Differences in immunomodulatory effects of hydrolysates might be explained by differences in TLR receptor activating properties, since some studies suggested an interaction between TLRs, mainly TLR2 and 4, and varying hydrolysates [37,49]. In accordance with the fact that whey and its hydrolysates with the lowest DH induced the highest levels of cytokine production by primary PBMCs, also higher TLR activation was observed in the HEK TLR screening assay. This TLR activation was lost in more extensively hydrolyzed whey protein. Neither intact casein nor casein hydrolysates activated TLRs. This suggests that the TLR interacting effects are both protein type (whey vs casein) and hydrolysis level dependent. Multiple TLRs were involved in the activating effects of whey (hydrolysates). Interestingly, the two mildest hydrolyzed whey samples, although inducing similar TLR activation in THP-1 cells, still showed distinct levels of cytokines produced. This could be explained by the fact that hydrolysates with a similar DH were found to interact with different TLR types (Figs 3 and 4). This suggests that small differences in the production process of hydrolysates may induce different peptides with specific receptor interaction properties. This also holds true for the whey hydrolysate with DH 15%, which induced stronger TLR activation compared to the other more hydrolyzed samples. However, until now the relation between receptor binding properties and specific steps in hydrolysis, including type of enzymes, temperature and time, is hardly studied. This would be an interesting and new topic for future research.

Besides the TLR activating capacity of the hydrolysates, also the inhibiting capacity of the hydrolysates was tested as potential mechanism for immunomodulation. In contrast to the lack of TLR stimulation by casein and its hydrolysates, these samples showed abundant TLR inhibiting effects. Whey hydrolysates, which showed potent TLR activating properties, on the other hand, only minimally inhibited TLRs. Intact whey only inhibited TLR3, while the whey hydrolysate with a DH of 7% inhibited TLR5 and 8. We did not detect inhibition of TLR4, which is the only inhibitory effect of a whey hydrolysate reported in literature [37]. This may be due to different experimental methods, such as the use of different cell lines or a different read-out system. The fact that only inhibitory effects of hydrolysates on TLR4 have been studied before, indicates that TLR inhibition has only recently been recognized as an important mechanism in immune regulation.

Overall, we showed that many hydrolysates were able to induce immunomodulatory effects and both activate and inhibit TLR signaling pathways. This indicates that specific protein sequences, either directly formed during hydrolysis in the intestine or added to nutrition after manufacturing, are able to actively stimulate immune activation and mature and modulate the immune system by TLRs. Furthermore, we also observed that specific immunomodulatory effects depend on differences in protein source and hydrolysis procedures used.

This knowledge is important for a better understanding of the effects of hydrolysates on the immune system and for the design of infant formulas, and nutrition in general, with specific immunomodulatory effects. Although in general more studies are needed to evaluate the effects of immune regulating peptides *in vivo*, our data suggest that addition of specific peptides to infant formulas can contribute to development of immunity in addition to the more traditional view of serving as nutritional source [37,50].

## Supporting information

S1 Fig Dose dependent NF-kB/AP-1 expression in THP-1-MD2-CD14 reporter cells after stimulation with graded loads of intact whey proteins or hydrolysates. Intact whey proteins and almost all whey hydrolysates induced a dose dependent increase in TLR activation in THP-1 reporter cells. Only the whey hydrolysate with a degree of hydrolysis of 22% did not show TLR activation. Statistical significant differences compared to the negative control were determined by using the Kruskal-Wallis test followed by the Dunn’s test and indicated by *.
(TIF)

S2 Fig Dose dependent NF-kB/AP-1 expression in HEK-Blue human TLR2 reporter cells after stimulation with graded loads of intact whey proteins or hydrolysates. Intact whey proteins and almost all whey hydrolysates induced a dose dependent increase in TLR activation in HEK hTLR2 reporter cells. Only the whey hydrolysate with a degree of hydrolysis of 22% did not show TLR2 activation. Statistical significant differences compared to the negative control were determined by using the Kruskal-Wallis test followed by the Dunn’s test and indicated by *.
(TIF)
