# Supplementary figures and images for: Toll-like receptor mediated activation is possibly involved in immunoregulating properties of cow's milk hydrolysates
Source: PLoS One. 2017 Jun 8;12(6):e0178191. doi: 10.1371/journal.pone.0178191 (PMC5464564; doi:10.1371/journal.pone.0178191)

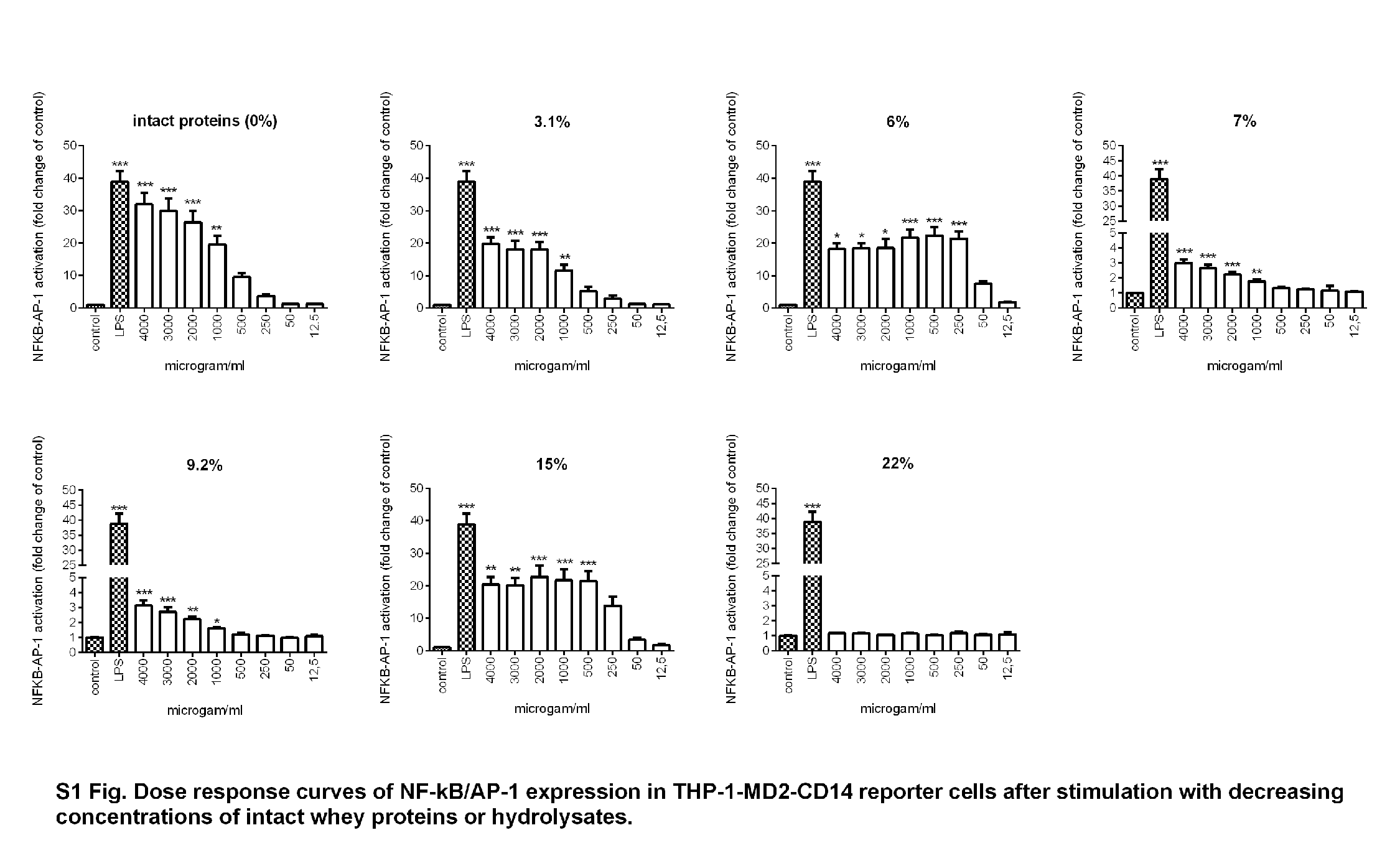

Supplement: S1 Fig — Intact whey proteins and almost all whey hydrolysates induced a dose dependent increase in TLR activation in THP-1 reporter cells. Only the whey hydrolysate with a degree of hydrolysis of 22% did not show TLR activation. Statistical significant differences compared to the negative control were determined by using the Kruskal-Wallis test followed by the Dunn’s test and indicated by *. (TIF) [file pone.0178191.s001.tif]

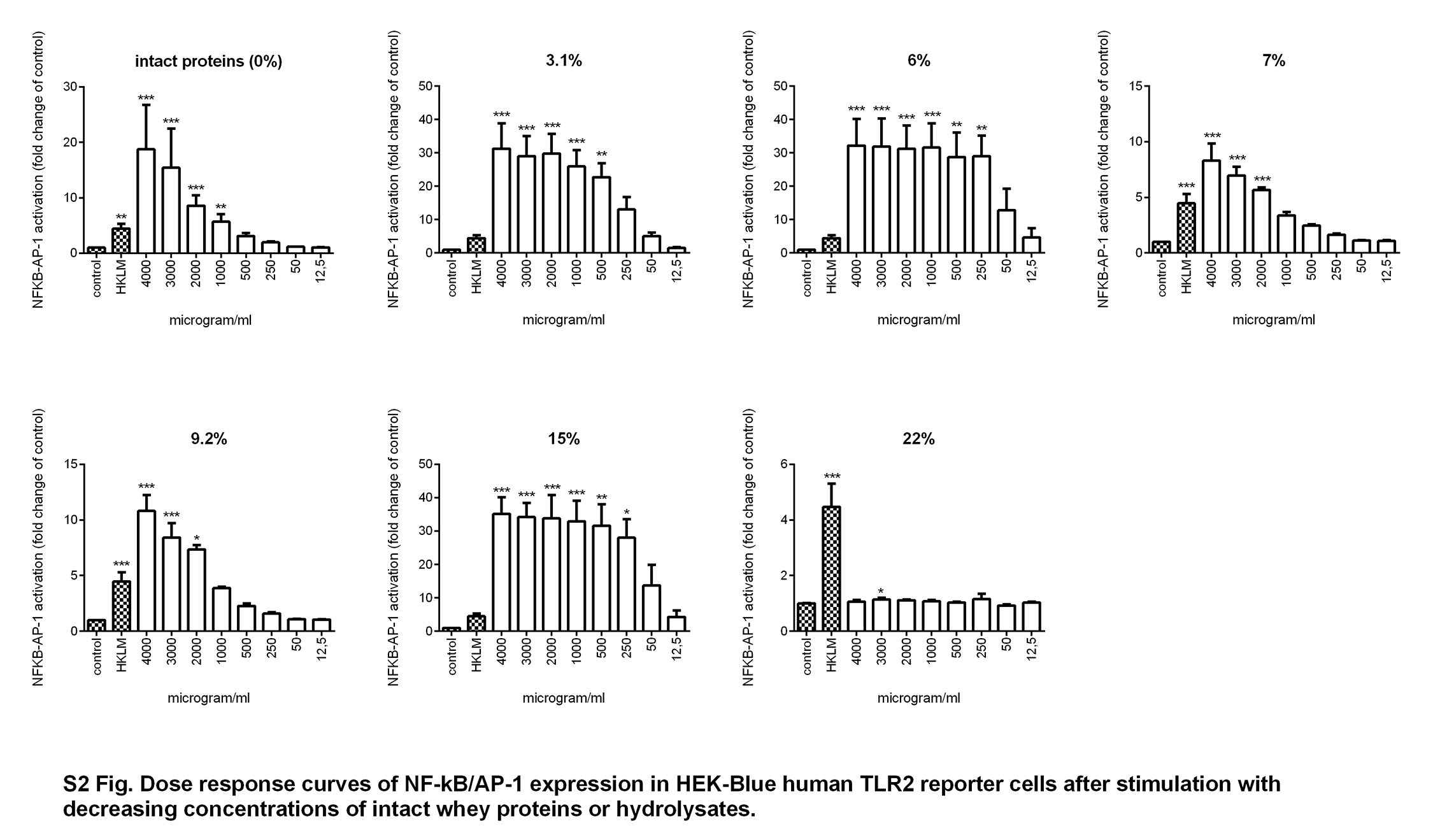

Supplement: S2 Fig — Intact whey proteins and almost all whey hydrolysates induced a dose dependent increase in TLR activation in HEK hTLR2 reporter cells. Only the whey hydrolysate with a degree of hydrolysis of 22% did not show TLR2 activation. Statistical significant differences compared to the negative control were determined by using the Kruskal-Wallis test followed by the Dunn’s test and indicated by *. (TIF) [file pone.0178191.s002.tif]
